# Inter-individual differences in foraging tactics of a colonial raptor: consistency, weather effects, and fitness correlates

**DOI:** 10.1186/s40462-020-00206-w

**Published:** 2020-06-24

**Authors:** Jacopo G. Cecere, Federico De Pascalis, Simona Imperio, Delphine Ménard, Carlo Catoni, Matteo Griggio, Diego Rubolini

**Affiliations:** 1 Area Avifauna Migratrice, Istituto Superiore per la Protezione e la Ricerca Ambientale (ISPRA), via Ca’ Fornacetta 9, I-40064 Ozzano dell’Emilia, BO Italy; 2 Dipartimento di Scienze e Politiche Ambientali, Università degli Studi di Milano, via Celoria 26, I-20133 Milan, Italy; 3 Ornis italica, piazza Crati 15, I-00199 Rome, Italy; 4 Dipartimento di Biologia, Università degli Studi di Padova, via U. Bassi 58/B, I-35131 Padova, Italy

**Keywords:** Dynamic foraging, Behavioural plasticity, Birds of prey, *Falco*, Foraging in flight, Foraging strategy, ODBA, Sit-and-wait

## Abstract

**Background:**

Consistent inter-individual differences in behavioural phenotypes may entail differences in energy efficiency and expenditure, with different fitness payoffs. In colonial-breeding species, inter-individual differences in foraging behaviour may evolve to reduce resource use overlap among conspecifics exploiting shared foraging areas. Furthermore, individual differences in foraging behaviour may covary with individual characteristics, such as sex or physiological conditions.

**Methods:**

We investigated individual differences in foraging tactics of a colonial raptor, the lesser kestrel (*Falco naumanni*). We tracked foraging trips of breeding individuals using miniaturized biologgers. We classified behaviours from GPS data and identified tactics at the foraging trip level by cluster analysis. We then estimated energy expenditure associated to each tactic from tri-axial accelerometer data.

**Results:**

We obtained 489 foraging trips by 36 individuals. Two clusters of trips were identified, one (SF) characterized by more static foraging behaviour and the other (DF) by more dynamic foraging behaviour, with a higher proportion of flying activity and a higher energy expenditure compared to SF. Lesser kestrels showed consistent inter-individual differences in foraging tactics across weather condition gradients, favouring DF trips as solar radiation and crosswind intensity increased. DF trips were more frequent during the nestling-rearing than during the egg incubation stage. Nestlings whose tracked parent was more prone to perform DF trips experienced higher daily mass increase, irrespective of nestling feeding rates.

**Conclusions:**

Our study provided evidence that breeding lesser kestrels flexibly adopted different foraging tactics according to contingent weather landscapes, with birds showing consistent inter-individual differences in the tendency to adopt a given tactic. The positive correlation between the tendency to perform more energy-demanding DF trips and nestling growth suggests that individual differences in foraging behaviour may play a role in maintaining key life-history trade-offs between reproduction and self-maintenance.

## Background

Inter-individual differences in behavioural phenotypes, that are consistent over time and across environmental contexts, have been frequently documented in animals [57]. Individuals can also consistently differ in how they modulate specific behaviours in accordance to spatial and temporal environmental gradients, the so-called contextual plasticity [64]. Ultimately, inter-individual differences in behavioural phenotypes and in contextual plasticity may be associated with fitness differences among individuals [63], with far-reaching ecological and evolutionary consequences [16]. For instance, individual differences in foraging behaviour may favour foraging specialization, resulting in a reduction of intraspecific competition by limiting resource use overlap [3, 8, 46].

In colonial species, where conspecifics gather in groups to reproduce close to each other and exploit shared foraging areas [11, 38], resource depletion around colony sites commonly occurs [4, 22]. Such depletion may favour the evolution and maintenance of individual foraging differences, which may be important in compensating the negative fitness effects of intraspecific competition. Indeed, individual differences in dietary preferences have been documented in several colonial vertebrates, including birds (e.g. [12]), pinnipeds (e.g. [14]) and terrestrial mammals (e.g. [15]). Individual foraging differences can also result in inter-individual differences in foraging tactics [5, 9, 17, 29], which we define as a distinguishable combination of behavioural patterns (i.e. multiple behaviours) shared by different individuals to search for food (similarly to [17, 39]). In northern gannets *Morus bassanus*, the analysis of both food boluses and blood isotopes, combined with the analysis of at-sea foraging behaviour, has uncovered individual differences in foraging tactics, with some birds exploiting consistently and more frequently than others fishing vessels discards [65]. In the same species, the analysis of foraging trips of birds breeding in two large colonies revealed consistent individual preferences in foraging areas during the nestling-rearing stage, but also large inter-individual differences in prey searching behaviour along environmental gradients [48]. Within-population differences in foraging behaviour can also be unrelated to individual preferences and rather arise from individual characteristics, such as sex [49], age [26], reproductive stage [25], personality [50] or physiological status differences [2].

The lesser kestrel *Falco naumanni* is a small (ca. 120 g) colonial diurnal raptor, which mostly breeds in holes and crevices of buildings in towns and cities, and forages in farmland areas surrounding breeding sites [11]. The species shows flexible foraging behaviour, whereby both flight and hunting mode vary in accordance to weather conditions: energy-saving soaring-gliding flight is more frequently adopted than energy-expensive flapping flight when solar radiation is high, and perch-hunting is more frequently used than flight-hunting when both wind speed and solar radiation are low [30]. However, it is as yet unknown whether individuals consistently differ in their foraging tactic across weather condition gradients, or whether individuals differ in their behavioural response to weather conditions.

In this study, we characterized the foraging tactics adopted by breeding lesser kestrels that were tracked by miniaturized biologgers (including both a GPS and a tri-axial accelerometer) across multiple foraging trips. We classified behaviours from movement data and identified tactics at the foraging trip level based on the combination of different behaviours occurring within each trip, while energy expenditure associated to each tactic was estimated by accelerometer data. According to previous knowledge about lesser kestrels foraging ecology [30], we expected birds to adopt two main foraging tactics: a more energy-demanding tactic whereby birds mainly search for prey while flying within a foraging trip (dynamic foraging, DF), and a less energy-demanding tactic, resulting from trips with prolonged perching while waiting for prey detection (static foraging, SF).

We then investigated 1) whether there were inter-individual differences in the tendency to adopt a given foraging tactic across weather condition gradients (solar radiation, rain, wind), expecting birds mainly to adopt the DF tactic whenever conditions are favourable for soaring-gliding, i.e. with high solar radiation [30] and wind assistance (tailwind or crosswind) at trip departure (e.g. [35]). Furthermore, we explored 2) whether the tendency to adopt a specific tactic was explained by individual characteristics, such as sex and breeding stage (incubation or nestling-rearing). Foraging behaviour of lesser kestrels can indeed vary markedly between males and females and between the incubation and nestling-rearing stages [31]. Finally, we investigated 3) the association between the tendency to perform a specific tactic and fitness-related traits. We expected the more energy-demanding DF tactic to be adopted preferentially by individuals in better body condition and to be associated with improved fitness, as estimated by higher breeding success and larger nestling body mass increase.

## Methods

### Target species, study area and general field methods

The lesser kestrel is a sexually dimorphic species, females being ca. 15% heavier than males [54]. Females lay up to 5 eggs that both parents incubate for ca. 30 days. After hatching, both parents feed the nestlings until fledging, which takes place at 35–40 days of age. The study was carried out in the city of Matera (southern Italy; 40°39′ N, 16°36′ E), hosting a colony of ca. 1000 breeding pairs [37]. We relied on nest-boxes placed on terraces of buildings in the old town, which were monitored 2–3 times per week to obtain detailed data about reproductive stage (laying date, incubation, hatching, nestling body mass at ca. 7 and ca. 14 days after hatching of the first egg) [53, 54]. Breeding individuals were captured by hand within nestboxes during the late incubation and early nestling-rearing stage. Upon capture, birds were individually marked, and body mass (using an electronic scale, accuracy 0.1 g) and keel length (using a dial calliper, accuracy 0.1 mm) were recorded.

### GPS deployment and identification of foraging trips

We equipped 36 breeding lesser kestrels (13 females and 23 males) with Axy-Trek biologgers, including a GPS and a tri-axial accelerometer (TechnoSmArt Europe S.r.l., Rome, Italy), using a backpack Teflon harness. Devices (including the harness) weighed between 5.9 and 7.2 g, on average accounting for 4.5% of body mass (range: 3.8–5.5%) (hereafter, relative load of device). The accelerometer was set to record data at 25 Hz and the GPS to record one position per minute from 05:00 to 21:00 local time (i.e. ca. 20 min before sunrise and ca. 30 after sunset; devices were switched off during night-time to preserve battery power). Birds were tagged in the morning and devices were set to start the following day in order to collect data when the tagged birds were likely inured to the device. After 2–5 days, birds were recaptured and the device was removed. Movement data were collected during June 1–20 of the breeding seasons 2016, 2017 and 2018, when pairs were in the late incubation or early nestling-rearing stage. Only one member of each pair was tracked and none of the birds was tagged more than once.

Foraging trips were identified as those tracks starting and ending within a 50-m buffer around the nest or the roosting site, and heading to the rural surroundings, by means of ESRI ArcMap 10.2.1. Since the devices switched on at 05:00 local time, in some cases the first position of the first foraging trip of the day was already in the countryside surrounding the town; when the distance between such position and the nesting site was > 2 km, the foraging trip was discarded and not included in any analysis. We did not consider as foraging trips all those excursions which covered the urban area only, identified by means of the 2012 CORINE Land Cover (CLC) map (codes 111 and 112, respectively continuous and discontinuous urban habitat), because birds generally do not forage in the urban area (authors’ pers. obs.). Each trip was classified as occurring during incubation if only eggs were present in the nest of the target individual on the date when the foraging trip was performed, or as occurring during the nestling-rearing stage if at least one nestling was present in the nest on the date when the trip was performed.

### Identification and characterization of foraging tactics

To identify behaviours adopted by lesser kestrels during foraging trips, we applied the Expectation Minimization binary Clustering (EMbC) algorithm to GPS data by means of the R package “EMbC” [27]. The EMbC is a classification algorithm based on maximum likelihood which assigns a behavioural mode to each GPS position according to instantaneous velocity and turning angle between successive positions. The algorithm assigns positions to four behavioural modes (see Fig. S1): 1) low velocity and low turns, which we interpreted as ‘perching’ behaviour (see Fig. S2); low velocity and high turns, representing ‘intensive search’; 3) high velocity and low turns, representing ‘relocation’; 4) high velocity and high turns, representing ‘extensive search’ [27, 39]. As the EMbC algorithm disregards the temporal information, we accounted for the possible incorrect labelling of positions when a long-term predominant behavioural mode occurred by applying a post-processing smoothing using the *smth()* function (with default parameters) (see [27]; and Fig. S1).

To identify the foraging tactics, we applied a cluster analysis to the percentage of the four behaviours occurring in each trip [39]. Cluster analysis was performed with a K-means procedure by means of the R package “stats” [56]. The optimal number of clusters was assessed by means of the *NbClust* procedure from the R package *“*NbClust” [13], which computes 30 indexes for determining the optimal number of clusters, including the option of no clustering (one cluster only). It then suggests the best number of clusters based on the majority consensus rule. As the *NbClust* procedure identified two clusters as the best number, we applied the K-means algorithm with K = 2 over 10,000 iterations.

We then assessed whether the two identified trip clusters (trip types, hereafter) affected variation in spatio-temporal trip descriptors (trip duration, trip length, maximum distance from the nest site and tortuosity, i.e. ratio between total trip length and the maximum distance from the nest site [7];) by means of linear mixed models (LMMs), including individual identity as a random intercept effect to account for non-independence of trips performed by the same individual.

Based on tri-axis accelerometer data, we calculated the overall dynamic body acceleration (ODBA) for each foraging trip, smoothing total acceleration over 1 s [59, 68]. ODBA is considered a proxy of energy expenditure in birds [23, 68]. It positively correlates with O_2_ consumption rates and CO_2_ production in great cormorants (*Phalacrocorax carbo*) [68] and with heart rate in two vulture species (*Gyps fulvus* and *G. himalayensis*) [20]. We investigated whether energy expenditure was affected by trip type by means of a LMM including individual identity as a random intercept effect. Accelerometer data were available for 34 out of 36 tracked birds.

LMMs were fitted using the *lmer* function of the R package “lme4” [6]. Residuals did not significantly deviate from a normal distribution.

### Environmental factors affecting foraging tactics

To investigate the effect of environmental conditions on the tendency to perform different trip types, we first associated to each trip the following variables: 1) solar radiation (W/m^2^) at departure, which seems to be determinant for performing soaring-gliding flight [30]; 2) presence of rain during the trip (hereafter, ‘presence of rain’; 0 = rain absent; 1 = rain present), which we hypothesized may negatively affect the likelihood of performing foraging in flight. Rain was not considered as a continuous variable since it occurred in 14% of foraging trips only, and considering it as continuous would have resulted in a very skewed variable with an excess of zeroes; 3) tail-wind (TWC) and 4) cross-wind components (CWC), both of which are known to affect movement activity in soaring-gliding raptors [35]. To control for potential differences in foraging behaviour between foraging habitats, we also computed 5) the percentage of positions in arable lands (the main habitat used for foraging, see below) for each trip (hereafter, ‘time in arable lands’). Nestbox identity was not included in the models since all tagged birds belonged to different nests, with the exception of two nestboxes which were sampled twice in different years but they were occupied by different individuals.

Solar radiation and rain data were recorded at a weather station located at 8 km from the nest sites (Matera, Contrada Matinelle, 40°41′ N; 16°31′ E). Wind data (speed and direction) were recorded at a different weather station, located at 15 km from the nest sites (Grottole 40°37′ N; 16°26′ E). All weather data were recorded at hourly intervals, and were associated to the GPS position that was closest in time.

TWC and CWC were calculated for each trip based on the mean value of wind speed and direction (WS and WD respectively) at time of departure and at time of returning, and the direction of the trip (TD), as follows:

$$ TWC= WS\times \cos \left( TD- WD\right) $$

$$ CWC=\mid WS\times \sin \left( TD- WD\right)\mid $$

TD, which we assumed to reflect the direction of the goal area, was calculated as the angle between the N-S axis (directed northwards) and the position of the farthest point of the trip from the nesting site. Positive TWC values imply that a bird flew globally with tail-wind on its way out of the colony towards the foraging grounds, whereas negative TWC values indicate the opposite (outgoing flights with headwinds). Large CWC values mean that a bird flew on average with high side-wind during the foraging trip.

To calculate the proportion of time spent in arable lands during each foraging trip, we assigned all GPS positions, excluding those identified as relocation by the EMbC, to the corresponding habitat type from CLC by means of ESRI ArcMap 10.2.1. We pooled together those CLC habitat types that were similar in habitat and structure, obtaining 6 habitat classes: artificial landscape (continuous and discontinuous urban fabric, infrastructures, industrial areas), arable lands, permanent crops (tree plantations, olive groves, vineyards), grasslands (pastures and natural grasslands), heterogeneous agricultural areas (annual crops associated with permanent crops, complex cultivation patterns, agro-forestry areas), and wooded areas (forests and bushes). Each trip was then characterized by the percentage of positions occurring in each habitat class (time spent in each habitat). While foraging, birds spent most of the time in arable lands (median 70.6%, 25th – 75th percentiles: 23.3 – 94.1%), and time in arable lands was negatively correlated with time in grasslands (*r* = − 0.69, *n* = 489 trips), the second most frequently used habitat (median 0.0%, 25th – 75th percentiles: 0.0 – 25.0%).

The probability to perform a given trip type (0 = SF, 1 = DF) was modelled by means of binomial generalised linear mixed models (GLMMs), with solar radiation, TWC, CWC, and presence of rain as fixed predictors, controlling for time in arable lands, breeding stage, sex and sampling year. Individual identity was included as a random intercept effect to control for non-independence of prey searching behaviour performed by the same individuals. All predictors were standardized (mean = 0 and SD = 1). Because of size and morphological differences, sexes may differ in their behavioural response to environmental variables; we hence included in the initial model all two-way interactions between sex and each weather variable (solar radiation, TWC, CWC, and presence of rain). The final binomial GLMM was obtained after removing weak (95% CI of parameter estimates intersecting 0) interactions in a single step. GLMMs were not overdispersed (ϕ always < 1.05).

### Individual differences in foraging tactics and their correlates

The random intercept effect of the final binomial GLMM describes the extent to which individuals preferentially perform different trip types (i.e. whether foraging tactics can be regarded as an individual-specific trait). Intra-idividual consistency of the probability of performing DF trips was estimated as the proportion of variance explained by the random intercept effect, accounting for variance explained by fixed effects (adjusted repeatability, *R*_adj_). *R*_*adj*_ was computed using the observation-level variance obtained via the delta method [45] and significance was assessed by a likelihood ratio test [69].

To investigate individual differences in the behavioural response to environmental gradients (behavioural reaction norms), which represent the degree of contextual plasticity (see [18]), we ran four binomial GLMMs with trip type as the binary dependent variable and sex, breeding stage, sampling year and one weather variable (solar radiation, presence of rain, TWC, or CWC) at a time as predictors, while including an individual-level random slope effect for that weather variable. Random slope models were fitted by including in binomial GLMMs one weather variable at a time to avoid model overparametrization and lack of convergence [60]. Significance of the random slope effect was tested by a likelihood ratio test [69]. Models were not overdispersed (ϕ always < 1.05). All binomial GLMMs were fitted using the *glmer* function of the R package “lme4” [6].

The individual tendency to perform DF trips was expressed as the individual-level random intercept estimate (hereafter, individual intercept) from the final binomial GLMM, higher values implying a stronger tendency to perform DF trips. Individual intercepts were computed as the conditional modes of the random effect evaluated at the parameter estimates (a.k.a. Best Linear Unbiased Predictors in LMMs). Uncertainty of individual intercepts was estimated by a simulation approach (*n* = 10,000 simulations; see [36]), and expressed as the SD of the simulations, using the *REsim* function of the R package “merTools” [36].

We tested the associations between the individual tendency to perform DF trips by the tracked parent and several fitness proxies, namely body condition of the tracked individual, its breeding success, and the daily body mass increase (DBMI) of its nestlings during the early nestling-rearing stage. We also investigated the association of the tendency to adopt the DF tactic with feeding frequency (number of foraging trips/h during the tracking period) and with the relative load of device (mass of biologging device relative to body mass). The latter association was tested (separately for males and females) to assess the possible effects of device load on foraging behaviour. All these associations were tested by computing weighted correlation coefficients (*r*_w_), where the weighting variable was the inverse of the SD of the individual tendency. Such weighting should at least partly account for uncertainty of conditional modes, as advocated by Houslay and Wilson [32]. Weighted correlation coefficients were computed using the *weightedCorr* function of the R package “wCorr” [24]. Significance of *r*_w_ was tested by randomization, randomly shuffling variables 9999 times, and computing the probability of observing a more extreme value than the observed one [41].

To estimate body condition of tracked birds, we computed the scaled mass index (hereafter, SMI) (body mass scaled by a skeletal trait, in our case keel length [51]). SMI was calculated as in Podofillini et al. [54]. To remove heterogeneity in SMI related to sex (see [54]), we computed the residuals of a linear model of SMI with sex as a predictor (hereafter, residual SMI).

The breeding success of the tracked birds was estimated as the fate of the brood, which was either coded as failed (0 = no nestlings alive at 14 days) or successful (1 = at least one nestling alive at 14 days after hatching of the first egg).

Nestling DBMI was computed as the mean of the daily relative body mass increase among all nestlings of a brood *k* between ca. 7 (mean ± SD = 6.6 ± 1.8 days) and ca. 14 days (mean ± SD = 14.7 ± 1.7 days) from hatching, as follows:

$$ {\mathrm{DBMI}}_k=\frac{1}{m}\times \sum \limits_{j=1}^m\frac{1}{i}\times \left[\frac{{\mathrm{BM}}_{\mathrm{j},\mathrm{day}\left(n+i\right)}-{\mathrm{BM}}_{\mathrm{j},\mathrm{day}(n)}}{{\mathrm{BM}}_{\mathrm{j},\mathrm{day}(n)}}\right] $$

where BM is body mass of nestling *j*, *n* is the day post-hatching at which the first record of BM was taken and *i* is the number of days elapsed between the first and second measure of BM of a nestling, *m* is the brood size at day_(*n+i*)_ (range: 1–4). We assumed that nestling DBMI denoted the ability of parents to foster the growth of their offspring, and that large value of DBMI could be considered as a proxy for high parental investment and high nestling fitness. To remove heterogeneity among sampling years and different brood sizes on nestling DBMI, we computed the residuals from a linear model of nestling DBMI including year and brood size at day *n+i* as predictors (hereafter, residual nestling DBMI). The correlation test was based on data from 22 parents whose eggs hatched and whose offspring were alive at 14 days from hatching of the first egg.

Feeding frequency was computed only for birds which were tracked during the nestling-rearing stage (*n* = 14). To remove heterogeneity among sampling years, sexes, and variation in brood size on feeding frequency (see [19, 31]), we computed residuals from a linear model of feeding frequency including year, sex, and brood size during the tracking days as predictors (hereafter, residual feeding frequency).

All analyses were ran on R ver 3.6.2 [56].

## Results

### Identification and characterization of foraging tactics

**Fig. 1:**
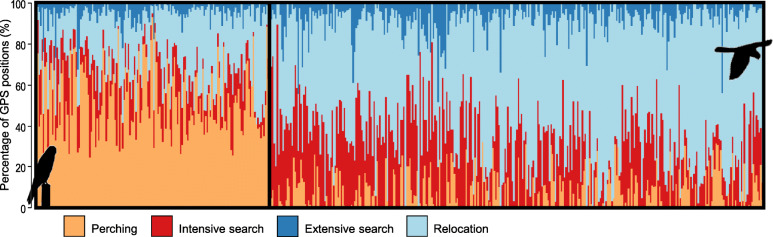
Percentages of GPS positions assigned to four behaviours (perching, intensive search, extensive search, relocation) within each trip (n = 489 trips); these four behaviours were derived from the behavioural modes assigned to GPS positions by the EMbC algorithm (see Methods). Black rectangles delimit the two clusters of trips identified by the cluster analysis, likely representing two foraging tactics (left cluster: 157 trips, static foraging trips; right cluster: 332 trips, dynamic foraging trips)

**Fig. 2:**
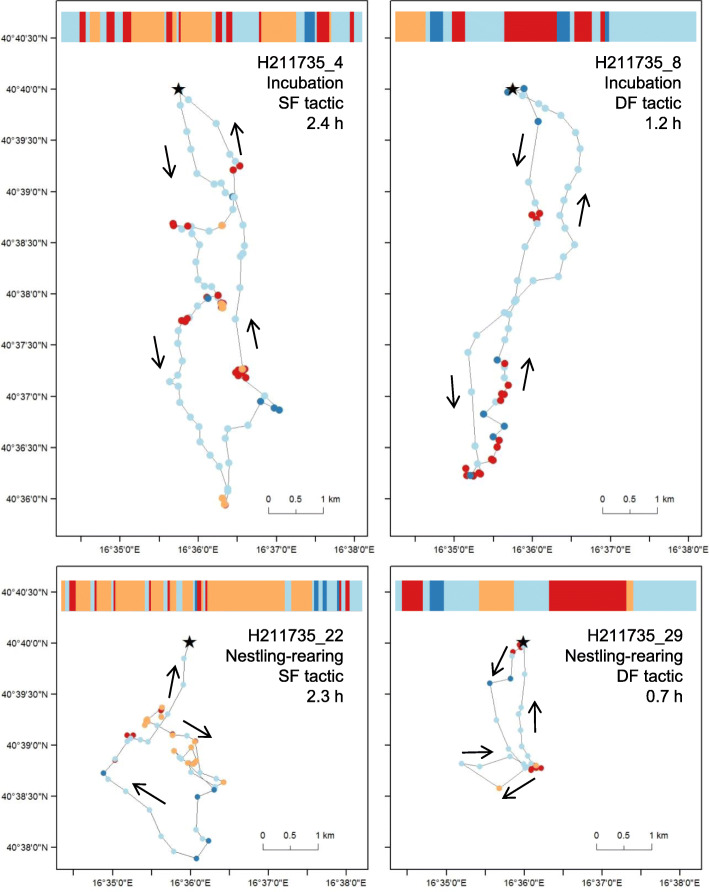
Representative examples of foraging trips identified as static (SF) (left panels) or dynamic foraging (DF) (right panels) performed by the same individual (H211735) during both incubation and nestling-rearing stages. Colours represent behaviours: perching (yellow), intensive search (red), relocation (light blue) and extensive search (dark blue). Identifier, breeding stage, tactic and duration of each trip are reported. Perching positions always represent multiple consecutive 1-min GPS-positions with same location, as shown by the band at the top of each panel depicting the temporal sequence of behaviours of the trip. Black star denotes nest site position, arrows the directions of movements

**Fig. 3:**
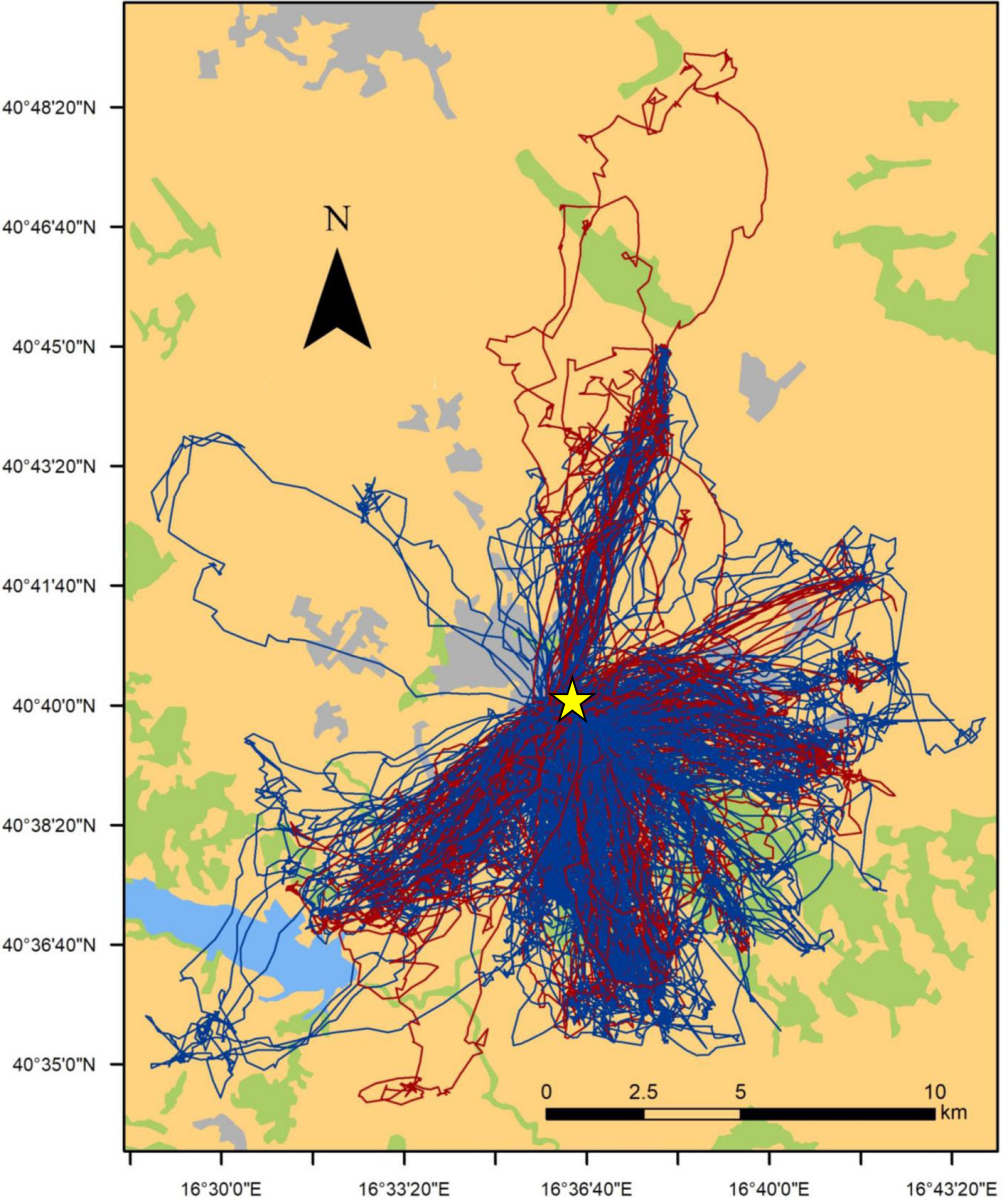
Map of 489 foraging trips from 36 lesser kestrels breeding in the city of Matera (southern Italy). Red lines: static foraging (SF) trips; blue lines: dynamic foraging (DF) trips; yellow star: location of breeding site of tracked individuals. Polygon colours on the background represent habitat types: urban areas (grey), farmland (dark yellow), semi-natural grasslands and woodlands (green), and water bodies (light blue)

We obtained 489 foraging trips from 36 breeding birds, the mean value being 14 trips (± 11 SD) per individual, ranging from a minimum of 1 to a maximum of 45 trips per individual (see also Table 1). The cluster analysis identified two clusters of trips, which we interpreted as two main foraging tactics (Fig. 1). The first cluster included trips characterized by high frequency of perching (mean ± SD proportion over all GPS positions of a trip: 0.53 ± 0.17), low frequency of intensive (0.15 ± 0.01) and extensive search (0.04 ± 0.05), and lower relocation positions (0.28 ± 0.12) compared to the other cluster. This cluster of trips was likely reflecting a relatively more SF tactic (Table 2). The second cluster was characterized by trips with a more dynamic and exploratory behaviour, with birds mostly searching for food while flying (perching: 0.07 ± 0.09; intensive search: 0.26 ± 0.16; extensive search: 0.07 ± 0.07; relocation: 0.60 ± 0.01), likely reflecting a relatively more DF tactic (Table 2). On average, SF trips lasted longer and were associated to lower values of ODBA compared to DF ones, whereas all other trip descriptors were not markedly different (Table 2). Figure 2 shows representative examples of DF and SF foraging trips performed by a single individual during both the incubation and the nestling rearing stages. Differences in the temporal sequence of behaviours clearly highlight that DF trips were characterized by more time actively spent searching for food, whereas SF trips showed prolonged perching periods (Fig. 2). There was no apparent spatial differentiation in exploited areas between SF and DF trips (Fig. 3).

**Table 1:** Variation in spatio-temporal trip descriptors according to breeding stage (incubation and nestling-rearing) and sampling year (2016, 2017 and 2018)

	**Trip duration (h)**	**Trip length (km)**	**Maximum distance (km)**	**Tortuosity**
*Incubation*				
2016 (*n* = 76, 12)	2.40 ± 1.61 (0.21–9.76)	18.23 ± 9.78 (3.86–55.59)	5.26 ± 2.46 (0.90–17.62)	3.49 ± 0.94 (2.25–5.96)
2017 (*n* = 91, 11)	2.02 ± 1.54 (0.37–8.61)	22.93 ± 12.40 (2.79–74.79)	6.63 ± 2.51 (0.61–13.33)	3.44 ± 1.11 (2.20–8.69)
2018 (*n* = 52, 6)	1.72 ± 1.33 (0.29–7.55)	18.14 ± 8.15 (6.07–39.58)	6.09 ± 1.58 (1.69–8.82)	2.90 ± 0.72 (2.13–4.70)
Years pooled (*n* = 219, 29)	2.08 ± 1.54 (0.21–9.76)	20.16 ± 10.83 (2.79–74.79)	6.03 ± 2.37 (0.61–17.62)	3.33 ± 0.10 (2.13–8.69)
*Nestling-rearing*				
2016 (*n* = 34, 2)	1.37 ± 0.84 (0.20–3.21)	14.68 ± 8.18 (3.21–29.34)	4.75 ± 2.44 (1.43–8.18)	3.11 ± 0.68 (2.19–4.83)
2017 (*n* = 78, 6)	1.09 ± 0.61 (0.25–2.93)	16.87 ± 7.37 (5.77–39.84)	6.03 ± 2.20 (2.45–13.98)	2.78 ± 0.51 (2.12–4.14)
2018 (*n* = 158, 6)	0.89 ± 0.54 (0.13–4.06)	15.42 ± 6.07 (3.34–35.78)	6.15 ± 2.33 (1.49–9.72)	2.56 ± 0.60 (2.06–5.30)
Years pooled (*n* = 270, 14)	1.01 ± 0.62 (0.13–4.06)	15.75 ± 6.77 (3.21–39.84)	5.94 ± 2.35 (1.43–13.98)	2.69 ± 0.61 (2.06–5.30)

For each variable, the mean value ± SD (minimum and maximum value) are reported. Sample sizes of both foraging trips and tracked birds are reported in the first column (7 individuals have been tracked during both breeding stages)

**Table 2:** Spatio-temporal descriptors and ODBA of static (SF) vs. dynamic foraging (DF) trips

**Variable**	**SF trips** **(*n*** **= 157)**	**DF trips** **(*n*** **= 332)**	**Estimate [95% CI]**	***F***	***df***	***P***	**Marginal** ***R***^**2**^	**Conditional** ***R***^**2**^
**Trip duration (h)**	**2.44 ± 1.54**	**1.04 ± 0.74**	**−1.31 [−1.52, −1.11]**	**156.7**	**1, 485**	**< 0.001**	**0.24**	**0.37**
Trip length (km)	18.22 ± 9.83	17.49 ± 8.72	− 0.72 [−2.46, 1.02]	0.65	1, 486	0.42	0.01	0.18
Maximum distance (km)	5.73 ± 2.50	6.10 ± 2.28	−0.01 [−0.43, 0.42]	0.01	1, 486	0.99	0.01	0.26
Tortuosity	3.19 ± 0.90	2.88 ± 0.83	−0.10 [−0.25, 0.06]	1.55	1, 485	0.21	0.01	0.30
**ODBA**^a^	**0.21 ± 0.08**	**0.42 ± 0.09**	**0.14 [0.13, 0.16]**	**292.7**	**1, 426**	**< 0.001**	**0.38**	**0.56**

The effect of trip type (SF = 0, DF = 1) on trip descriptors and ODBA was assessed by linear mixed models including individual identity as a random intercept effect. Marginal (proportion of variance explained by fixed effects) and conditional (proportion of variance explained including both fixed and random effects) *R*^2^ were estimated by means of the R package “performance” [44]. Mean values ± SD are reported. Degrees of freedom for the *F*-tests were estimated according to the Kenward-Roger approximation. Important effects (whose 95% CI do not include zero) are bolded

### Environmental factors affecting foraging tactics and individual differences in foraging behaviour

**Fig. 4:**
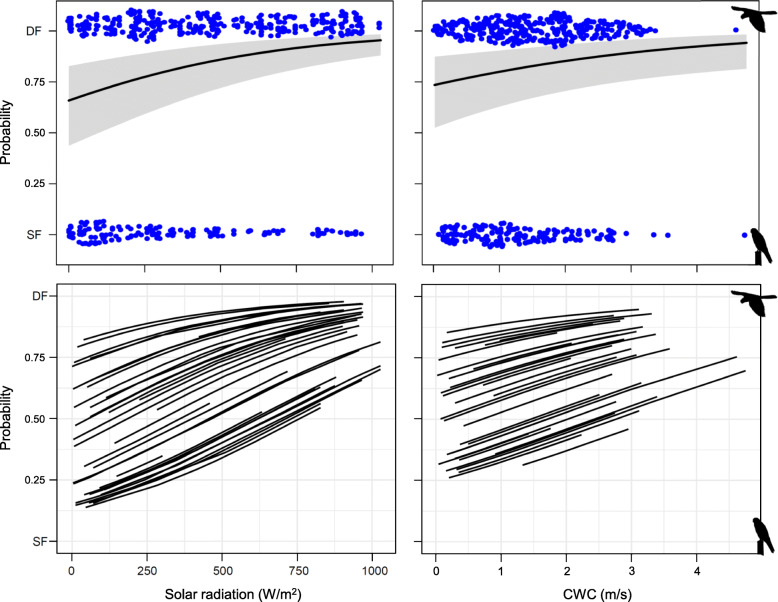
Population-level (upper panel) and between-individual (lower panel) variation in the probability of performing dynamic (DF) vs. static (SF) foraging trips according to solar radiation (W/m^2^) and CWC (cross-wind component, m/s), as estimated by the binomial GLMM reported in Table 3. Upper panel: partial regression plots (with 95% confidence bands) with dots representing actual trip types (‘sinaplot’ visualization [62]). Lower panel: model-predicted regression lines (random intercept, fixed slope) for 35 individuals included in the analyses

Tracked birds preferentially performed DF trips (65% of all trips; intercept-only binomial GLMM, estimate [95% CI] = 0.62 [0.18, 1.06], *Z* = 2.76, *P* = 0.006). The probability of performing DF trips varied among years and was moderately positively affected by solar radiation (effect size *r* = 0.28), with CWC and breeding stage having somewhat weaker effects (*r* = 0.13 and 0.20, respectively) (Table 3). With low CWC and solar radiation, birds mostly adopted SF, whereas the probability of adopting the DF tactic increased as CWC and solar radiation increased (Fig. 4). Birds were more likely to perform DF trips during the nestling-rearing compared to the incubation stage (Table 3). All other predictors had a negligible effect on the probability to perform DF trips (95% CI including 0, all *r* <  0.06). Males and females did not markedly differ in the probability to perform DF trips according to weather condition gradients (two-way interactions between sex and each weather variable, *r* always < 0.05, *P* always > 0.29).

**Table 3:** Final binomial generalized linear mixed model of the probability to perform dynamic foraging (DF) trips over static foraging (SF) trips

**Predictors**	**Estimate [95% CI]**	**χ**^**2**^	***df***	***P***	**Effect size*r***
**Solar radiation**	**0.70 [0.44, 0.97]**	**26.88**	**1**	**< 0.001**	**0.28**
Presence of rain	−0.12 [−0.35, 0.11]	1.09	1	0.30	0.06
TWC	−0.16 [−0.44, 0.12]	1.27	1	0.26	0.06
**CWC**	**0.32 [0.08, 0.56]**	**6.90**	**1**	**0.009**	**0.13**
Time in arable lands	0.13 [−0.13, 0.38]	0.97	1	0.32	0.06
**Breeding stage**	**0.53 [0.19, 0.86]**	**9.33**	**1**	**0.002**	**0.20**
Sex	0.04 [−0.36, 0.44]	0.03	1	0.86	0.01
**Sampling year**^**a**^	**–**	**8.84**	**2**	**0.012**	**–**
Intercept	−0.02 [−0.73, 0.69]				

^a^: estimated mean values (logit scale), LCL, UCL: 2016 = -0.179, -0906, 0.548; 2017 = 1.199, 0.377, 2.003; 2018 = 0.959, a 0.287, 1.631

Estimates refer to standardized variables. Breeding stage was coded as 0 = incubation or 1 = nestling-rearing, sex as 0 = males or 1 = females. Individual identity was included as a random intercept effect. The model was not overdispersed (ϕ = 1.0). Model *R*^2^ was 0.24 (marginal) and 0.34 (conditional), while *R*_adj_ was 0.13 (all values estimated according to [45]). Effect size for covariates was calculated as the absolute value of Pearson’s *r* obtained from semi-partial *R*^2^ values from the “r2glmm” R package [34]. Important effects (i.e., with 95% CI of estimates not including zero) are shown in bold. One individual with a single foraging trip was excluded (*n* = 488 trips from 35 individuals)

The proportion of variance explained by individual identity was low (*R*_adj_ = 0.13, χ^2^ = 15.17, *df* = 1, *P* < 0.001), indicating that individuals mostly adopted a flexible foraging behaviour. However, variation among individuals in the tendency to perform DF trips was substantial, with values ranging between −1.15 and 1.65 (logit scale; Fig. S3). Individual differences in the behavioural response to weather conditions gradients were negligible in most cases (random slope effects, solar radiation: χ^2^ = 0.09, *df* = 2, *P* = 0.96; presence of rain: χ^2^ = 2.02, *df* = 2, *P* = 0.36; TWC: χ^2^ = 5.60, *df* = 2, *P* = 0.06; CWC: χ^2^ = 1.10, *df* = 2, *P* = 0.58). Hence, individuals consistently differed in foraging tactic across solar radiation and CWC gradients (Fig. 4).

### Correlates of individual variation in foraging tactics

**Fig. 5:**
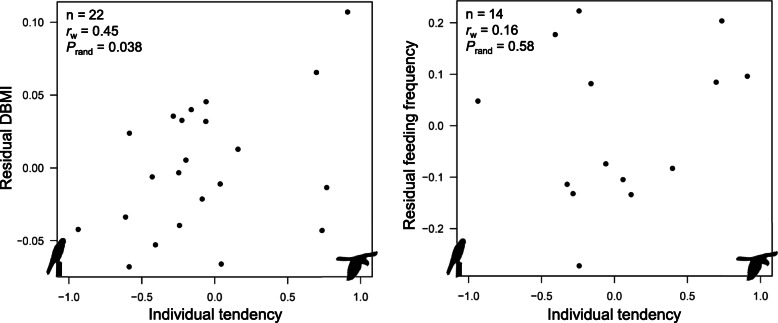
Association between the individual tendency to perform dynamic foraging (DF) trips and (left panel) residual nestling daily body mass increase (DBMI) or (right panel) residual feeding frequency (trips/hour). Sample size, weighted correlation coefficient *r*_w_ and *P*-value are reported within panels

The individual tendency to perform DF trips was very weakly correlated with residual SMI (*r*_w_ = −0.03, *n* = 35, *P*_rand_ = 0.87) and breeding success (*r*_w_ = 0.15, *n* = 35, *P*_rand_ = 0.41), but it was moderately positively correlated with the residual nestling DBMI (*r*_w_ = 0.45, *n* = 22, *P*_rand_ = 0.038) (Fig. 5). The body mass of nestlings increased on average by 11.4% (± 4.7 SD) between the two measurements, ranging between 4 and 29%. The latter correlation was not explained by a higher feeding frequency of birds performing more DF trips, as feeding frequency was weakly correlated with the tendency to perform DF trips (*r*_w_ = 0.16, *n* = 14, *P*_rand_ = 0.58) (Fig. 5).

Finally, the relative load of devices was very weakly associated with the individual tendency to perform DF trips in both sexes (males: *r*_w_ = 0.08, *n* = 23, *P*_rand_ = 0.71; females: *r*_w_ = − 0.07, *n* = 12, *P*_rand_ = 0.83).

## Discussion

By GPS-tracking individuals over multiple foraging trips, we investigated the degree of individual specialization in foraging tactics of breeding lesser kestrels. Foraging tactics adopted by birds during foraging trips varied between two extremes. On the one side, birds performed more static foraging (SF) trips, characterized by high frequency of perching and low proportion of searching behaviour, which were also long-lasting. On the other side, high frequency of both relocation and intensive search and low frequency of perching resulted in more dynamic foraging (DF) trips, that were short-lasting. Unsurprisingly, DF trips were associated with 1.7-fold higher ODBA compared to SF trips. Although birds performed more DF trips overall, the probability of performing DF trips increased with increasing solar radiation and crosswind, and was higher during the nestling-rearing than during the egg incubation stage. On top of this, individuals significantly differed in their tendency to perform different trip types, with no evidence of contextual plasticity (i.e. all individuals modulated their foraging tactic in a similar way in relation to weather condition gradients). The tendency to perform energy-demanding DF trips by the attending tracked parent was associated with higher nestling body mass increase, but not with a higher nestling feeding frequency.

The occurrence of both wide-ranging and relatively more static foraging tactics has been documented in many predators, including reptiles (e.g. [52]), fish (e.g. [21]), birds (e.g. [42]) and mammals [67]. However, to our knowledge, the alternation of foraging tactics has seldom been analysed at the individual level. It is being increasingly appreciated that animal movement patterns and behaviour are shaped by the so-called ‘energy landscape’, i.e. the variation in the cost of transport across time and space, determined by the interaction between static landscape features and dynamic environmental conditions [1, 23, 61]. Lesser kestrels heavily rely on thermal soaring and gliding for foraging, especially when solar radiation is high [30]. We showed that foraging lesser kestrels mainly performed SF trips when weather conditions were not ideal for soaring-gliding, i.e. with low solar radiation [30] and weak crosswinds, which are known to affect movement patterns in soaring raptors [35]. As expected according to previous studies [43, 66], performing SF trips was an energy-sparing tactic compared to performing DF trips. Birds may thus use DF or SF depending on the relative energy advantage, determined by the wind and solar radiation landscape surrounding the breeding colony. Birds were mostly adopting the more energy-demanding tactic (i.e., DF) only when the energy landscape surrounding breeding sites allowed it.

Inter-individual differences in foraging tactics of colonial vertebrates may originate from divergent selection to mitigate intraspecific competition, by limiting resource use overlap among individuals sharing foraging areas (e.g., [3]). Under this scenario, we would not expect any significant fitness difference between individuals adopting preferentially one or the other foraging tactic. Although we did not measure other fitness components (i.e., parental survival), the higher body mass increase of nestlings whose parent was performing more DF trips might suggest that the tendency to adopt a given foraging tactic is related to individual characteristics, such as age/experience or physiological status [2, 26] rather than to the mitigation of intraspecific competition. Admittedly, the higher body mass increase of nestlings whose parent was preferentially performing DF trips should be viewed with caution because we could assess the behaviour of a single parent only. Notwithstanding, it suggests that, even when considering the uniparental contribution to nestling growth, an increased energy expenditure during foraging could result in faster nestling growth and thus better fitness prospects.

The higher body mass increase of nestlings of tracked parents was not a by-product of higher nestling feeding frequencies of birds preferentially performing DF trips. This raises the question of why nestlings reared by parents more prone to perform DF trips grew more. We may speculate that parents preferentially performing DF trips could have delivered more energy-rich prey to their nestlings than those performing more SF trips, resulting in faster mass growth. For instance, parents performing more DF trips may have been mostly targeting large crickets, that are the preferred lesser kestrel prey and have a higher fat content compared to e.g. vertebrate preys [58], whereas those performing more SF trips may have been targeting larger (but less energetic) prey items, such as lizards and mammals. Indeed, sit-and-wait predators generally catch larger prey compared to those taken by active predators [28].

The shorter duration of DF compared to SF trips could be related to the DF tactic being associated with group foraging by means of local enhancement processes for food finding [55]. Such processes imply that individuals searching for food are attracted by feeding aggregations of other individuals and do not need to spend time searching for productive food patches [33, 40]. Social foraging should increase individual foraging efficiency when exploiting ephemeral and unpredictable resources [47]. In the study area, we indeed regularly observed aggregations of foraging lesser kestrels performing DF to catch large orthopterans flushed during harvesting operations (see also [10]), while birds perching on wires or poles were generally observed alone.

## Conclusions

We provided evidence for both individual foraging specialization and high flexibility in foraging tactics, with individuals consistently modulating their foraging tactic according to the concomitant weather landscape. The two foraging tactics were not equivalent in term of energy expenditure and consequences for fitness. Parents preferentially performing DF trips may have exploited group foraging to target more profitable, energy-rich prey in a shorter amount of time, resulting in increased nestling growth, though at the cost of a higher energy expenditure for transport. We may speculate that parents mainly performing DF trips may favour offspring growth over self-maintenance, whereas those mainly performing SF trips may do the opposite. Our results therefore suggest that inter-individual differences in foraging tactics may play a role in maintaining variation within populations in key intergenerational life-history trade-offs, such as those between parental reproductive effort and offspring survival, or between offspring growth and parental self-maintenance.

## Supplementary information

**Additional file 1. **Contains the scatterplot of GPS positions in relation to flight velocity and turning angle, highlighting the four behavioural modes assigned by the EMbC algorithm (**Figure S1.**), the frequency histograms of ODBA values associated to GPS positions for each behavioural mode obtained by the EMbC algorithm, showing that behavioural modes largely differ in ODBA values (**Figure S2.**), and the ‘caterpillar plot’ illustrating the variation of the random effect estimates obtained by simulations from the final binomial GLMM (**Figure S3.**).

## Data Availability

The datasets used and/or analyzed during the current study are available from the corresponding author on request.

## Abbreviations

BM: Body mass
CLC: CORINE Land Cover
CWC: Cross-wind component
DBMI: Daily body mass increase
DF: Dynamic foraging
EMbC: Expectation Minimization binary Clustering
GLMM: Generalised linear mixed model
LMM: Linear mixed model
ODBA: Overall dynamic body acceleration
SF: Static foraging
SMI: Scaled mass index
TD: Direction of the trip
TWC: Tail-wind component
WD: Wind direction
WS: Wind speed
