# Supplementary figures and images for: Inter-individual differences in foraging tactics of a colonial raptor: consistency, weather effects, and fitness correlates
Source: Mov Ecol. 2020 Jun 24;8:28. doi: 10.1186/s40462-020-00206-w (PMC7313117; doi:10.1186/s40462-020-00206-w)

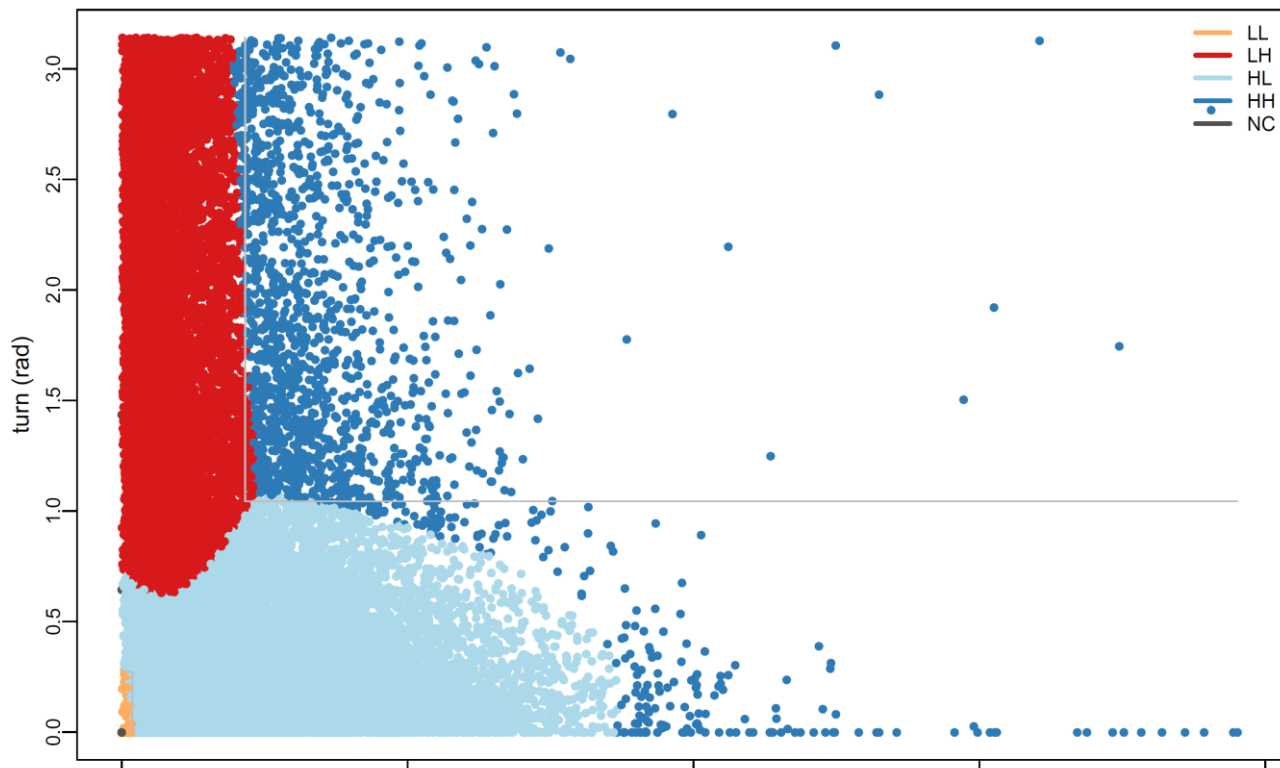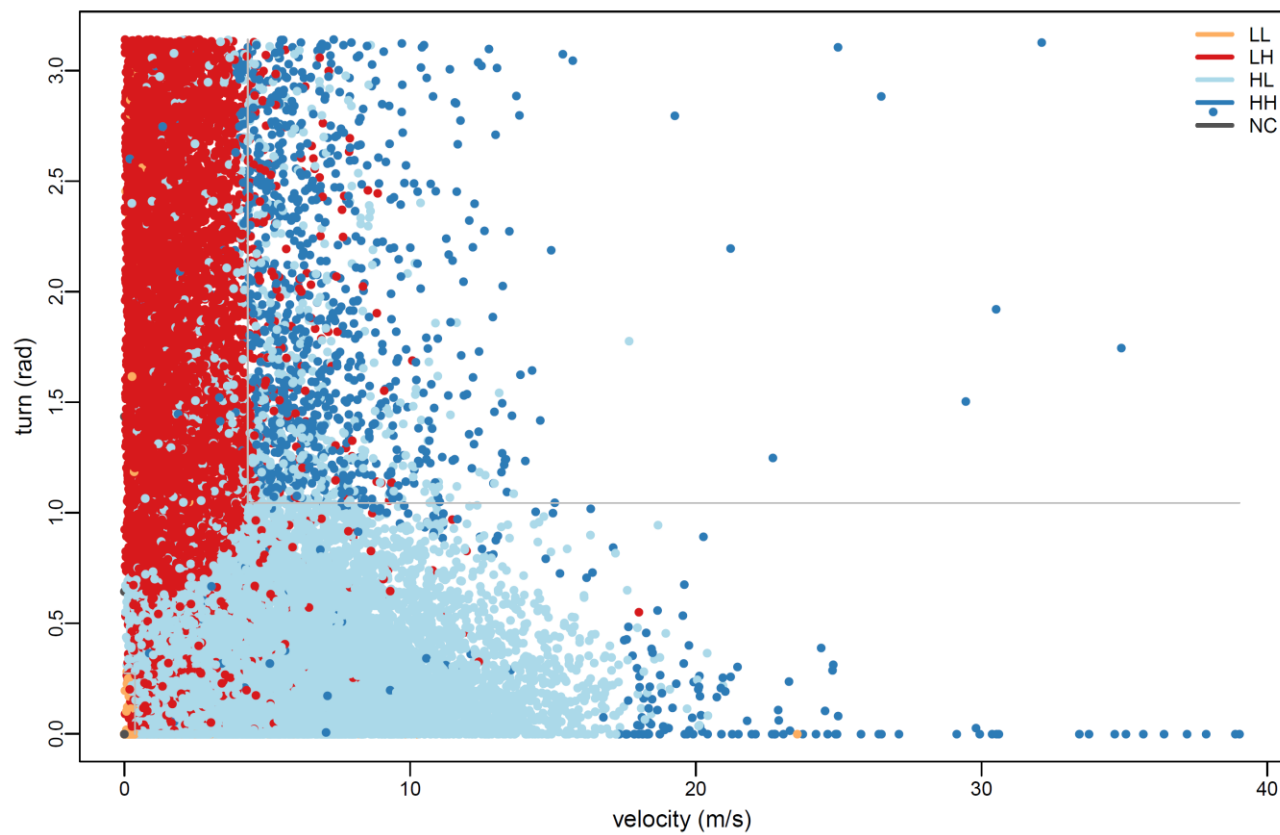

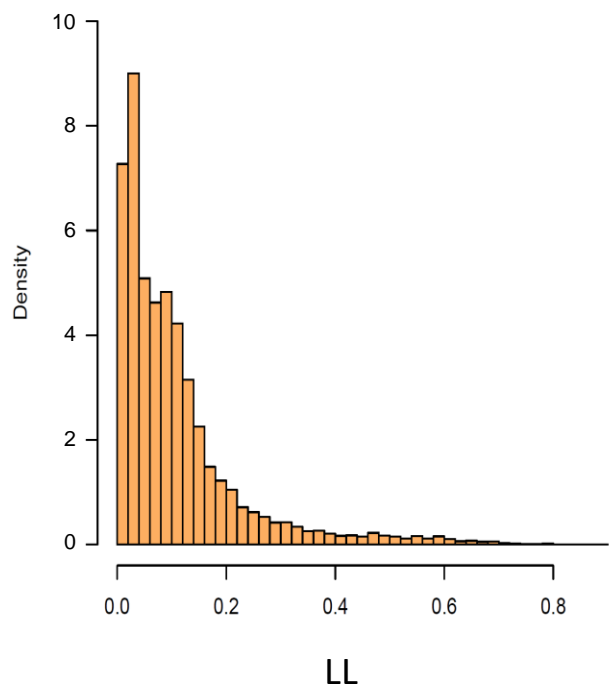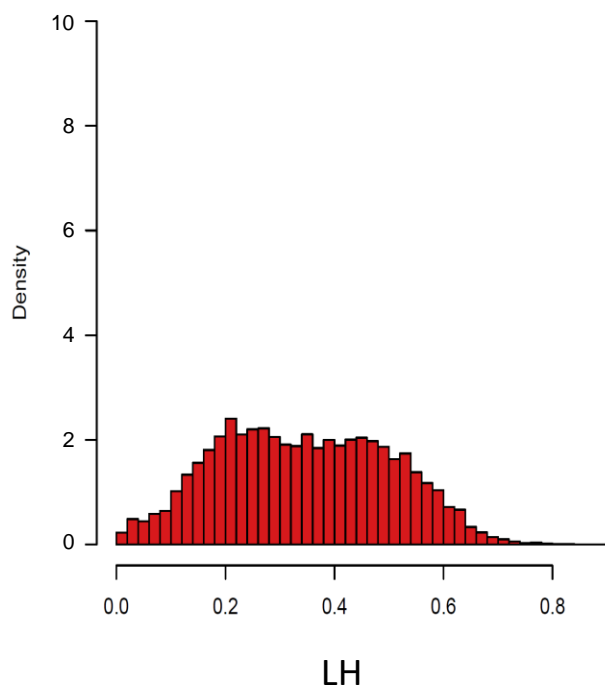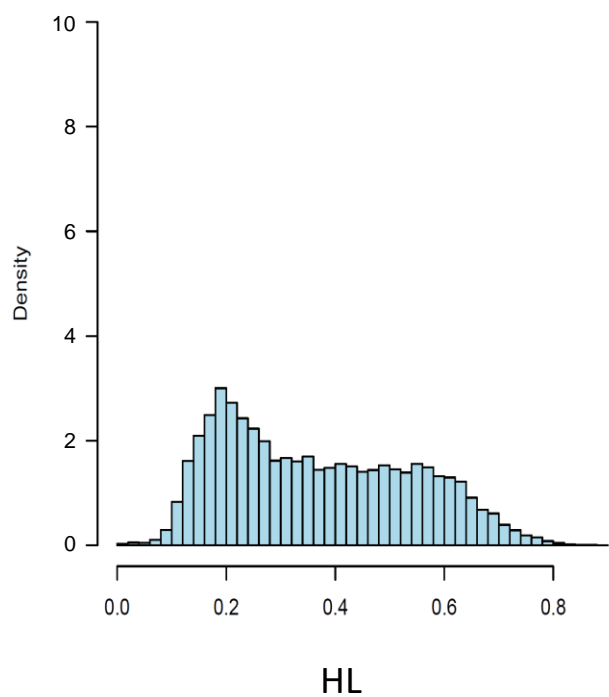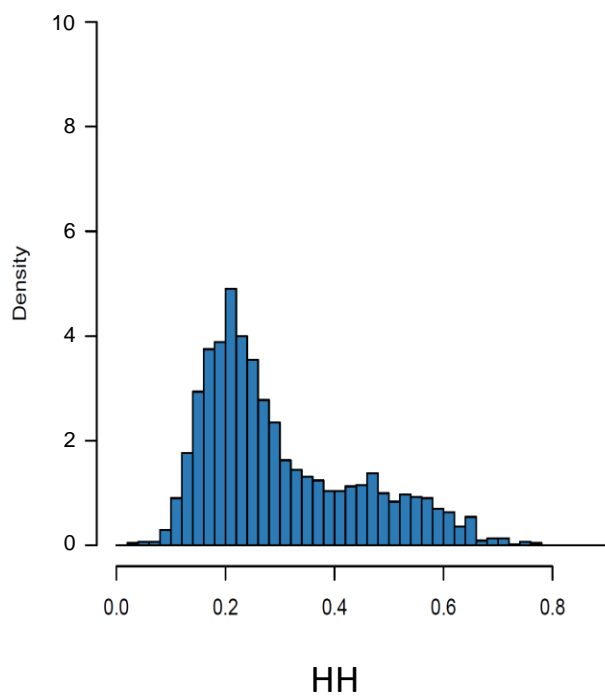

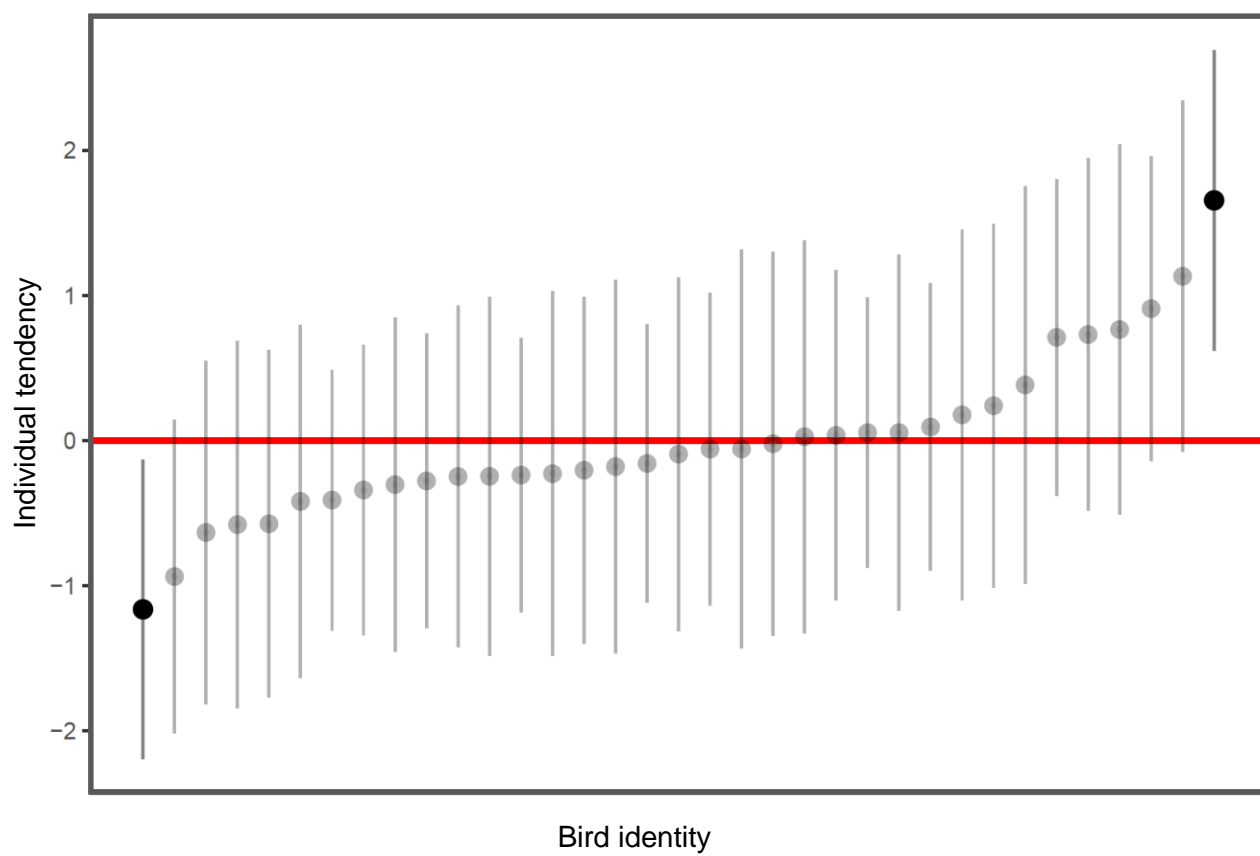

Supplement: Supplementary file 1 — Additional file 1. Contains the scatterplot of GPS positions in relation to flight velocity and turning angle, highlighting the four behavioural modes assigned by the EMbC algorithm (Figure S1.), the frequency histograms of ODBA values associated to GPS positions for each behavioural mode obtained by the EMbC algorithm, showing that behavioural modes largely differ in ODBA values (Figure S2.), and the ‘caterpillar plot’ illustrating the variation of the random effect estimates obtained by simulations from the final binomial GLMM (Figure S3.). [file 40462_2020_206_MOESM1_ESM.pdf]
